# Effects of mechanical stimulation on metabolomic profiles of SW1353 chondrocytes: shear and compression

**DOI:** 10.1242/bio.058895

**Published:** 2022-02-03

**Authors:** Hope D. Welhaven, Carley N. McCutchen, Ronald K. June

**Affiliations:** 1 Department of Chemistry & Biochemistry and Molecular Biosciences Program, Montana State University, Bozeman, MT 59717, USA; 2 Department of Mechanical & Industrial Engineering, Montana State University, Bozeman, MT 59717, USA; 3 Department of Microbiology & Cell Biology, Montana State University, Bozeman MT 59717, USA; 4 Department of Orthopaedics & Sports Medicine, University of Washington, Seattle, WA 98195, USA

**Keywords:** Chondrocyte, Mechanobiology, Mechanotransduction, Metabolomics, Osteoarthritis

## Abstract

Mechanotransduction is a biological phenomenon where mechanical stimuli are converted to biochemical responses. A model system for studying mechanotransduction are the chondrocytes of articular cartilage. Breakdown of this tissue results in decreased mobility, increased pain, and reduced quality of life. Either disuse or overloading can disrupt cartilage homeostasis, but physiological cyclical loading promotes cartilage homeostasis. To model this, we exposed SW1353 cells to cyclical mechanical stimuli, shear and compression, for different durations of time (15 and 30 min). By utilizing liquid chromatography-mass spectroscopy (LC-MS), metabolomic profiles were generated detailing metabolite features and biological pathways that are altered in response to mechanical stimulation. In total, 1457 metabolite features were detected. Statistical analyses identified several pathways of interest. Taken together, differences between experimental groups were associated with inflammatory pathways, lipid metabolism, beta-oxidation, central energy metabolism, and amino acid production. These findings expand our understanding of chondrocyte mechanotransduction under varying loading conditions and time periods.

This article has an associated First Person interview with the first author of the paper.

## Abstract (precis)

**Summary:** SW1353 chondrocyte-like cells respond to shear and compressive stimulation through changes in diverse metabolic pathways between 15 and 30 minutes of stimulation.

## INTRODUCTION

Mechanotransduction is the study of the cellular responses to applied mechanical loads and deformations (Paluch et al., 2015). In the past, mechanotransduction has been studied in sensory cells, such as hair cells showing intricate mechanisms of mechanosensing (Gillespie and Muller, 2009). Beyond sensory cells, the musculoskeletal system, consisting of bone, cartilage, muscle, tendons, and ligaments, functions to provide support, stability, and movement for the organism. In doing so, these tissues experience a variety of mechanical loads (compressive, tense, and shear) that, through cellular mechanotransduction, must be interpreted, dispersed, and transduced by resident cells. Physiological levels of loading are necessary for cell growth, proliferation, and survival in tissues like articular cartilage (AC) (Jaalouk and Lammerding, 2009; Orr et al., 2006).

AC provides near-frictionless articulation on the ends of long bones, which provides a smooth surface for joint movement, resists extreme loads that are applied to the joint, and reduces the stress placed on underlying bone (Sophia Fox et al., 2009). Chondrocytes synthesize an extracellular matrix (ECM), which is the main component of cartilage (Sanchez-Adams et al., 2014). The ECM performs mechanotransduction and helps to maintain homeostasis during mechanical loads. Therefore, under load associated with joint motion, the ECM is stimulated to support cartilage homeostasis. Although sub-injurious mechanical loading is required to maintain cellular homeostasis, loads large in duration and/or magnitude can lead to biological imbalances that disrupt homeostasis and induce joint disease such as osteoarthritis (OA) (Racunica et al., 2007).

OA is a chronic joint disease that is caused by many factors including inflammation and joint injury that results in the eventual breakdown of AC. Degradation of tissue ultimately leads to joint pain, limited mobility, and reduced quality of life. Therefore, investigation of mechanotransduction and metabolism of AC, chondrocytes, and the ECM may elucidate the effects of mechanical loading on the joint.

To this end, there has been substantial research in the field to expand our understanding of how chondrocytes respond to the mechanical stimuli experienced by the joint (Bricca et al., 2017; Chan et al., 2018, 2016). Building on these results, this study examined how chondrocytes respond to cyclical shear and compressive loading *in vitro* by utilizing untargeted metabolomics.

Metabolomics is an analytical profiling technique used to investigate large numbers of small molecule intermediates called metabolites. These molecules “act as a spoken language, broadcasting signals from the genetic architecture and the environment” (Jewett et al., 2006). Metabolomic profiling analyzes thousands of small molecules characterizing the cellular phenotype and provides an unbiased view of metabolic shifts induced by experimental conditions (Carlson et al., 2018). This technique is applicable to the study of chondrocyte metabolism and OA because it has the ability to provide metabolic information, such as involved pathways and intermediates, in response to applied stimuli, such as shear and compression. While many studies have investigated the effects of mechanical stimuli on the joint, few have investigated if mechanical stimuli impact the metabolism of involved tissues.

Much of what is known about cartilage's biosynthetic response, ECM and proteoglycan synthesis, and overall cartilage health has been learned through the lens of compressive stimuli. Modeling predicts that the chondrocyte pericellular matrix enables macroscale strain transfer directly to chondrocytes (Kim et al., 2008). Early *in vitro* studies demonstrated that cyclical compression of chondrocytes results in proteoglycan synthesis and deposition of pericellular matrix (Quinn et al., 1998). Ion channels TRPV4, Piezo1, and Piezo2 are involved in chondrocyte mechanotransduction due to the localized changes in ion concentrations that develop upon cartilage compression (Lee et al., 2014; O'Conor et al., 2014). *In vivo* studies suggest that cyclical cartilage compression associated with exercise is protective of chondrocytes. Bricca et al. varied doses of exercise and found that cyclical loading serves as a protective tool for chondrocytes to withstand loads that are increasing in magnitude and frequency (Bricca et al., 2017). In contrast, extended periods of increasing load can interfere with homeostasis leading to deterioration of the ECM, which can induce joint disease such as OA (Chan et al., 2018).

While numerous studies investigate compressive stimuli, fewer studies have examined shear as a mechanical stimulus. Early studies showed that shear stimulation of cartilage explants results in matrix synthesis and release of superficial zone protein in a TGFβ-dependent manner (Jin et al., 2001; Neu et al., 2007). Smith et al. compared shear stress and hydrostatic pressure on chondrocytes and determined that shear stress increased proinflammatory mediator release, nitric oxide production, and induced apoptosis molecular changes, but decreased matrix protein expression (Smith et al., 2004). Chih-Chang et al. studied regulation of chondrocyte urokinase plasminogen activator (uPA) expression by shear stress and determined shear stress is somewhat protective against uPA upregulation induced by OA (Yeh et al., 2013). The molecular switch, RhoA, regulates signaling cascades triggered by mechanotransduction. Wan et al. find that a range of shear stress magnitudes are important for RhoA activation, inhibition, and general function in chondrocytes (Wan et al., 2013). This is important in light of the seminal demonstration of *in vivo* shear and compressive strains in human cartilage (Chan et al., 2018, 2016).

Because of the previous demonstrations that mechanical stimuli can promote chondrocyte biosynthetic responses, our recent studies examined the effects of cyclical compression on chondrocyte metabolism (Jutila et al., 2014; McCutchen et al., 2017; Salinas et al., 2017, 2019; Zignego et al., 2014). Central metabolism is responsible for production of all non-essential amino acids, and these comprise a substantial fraction of cartilage matrix proteins. These studies suggest physiological dynamic compression in chondrocytes is primarily mediated by amino acid, lipid, and central energy metabolism (Zignego et al., 2015). Using targeted metabolomics, it appears that physiological cyclical compression results in changes in the distribution of the metabolites of central metabolism (Jutila et al., 2014; Salinas et al., 2017; Zignego et al., 2015). Stoichiometric modeling and metabolomic flux analysis suggest that 15 min of cyclical compression result in an initial burst of metabolic activity that is followed by metabolic recovery when chondrocytes are compressed for an additional 15 min.

Knowing that both shear and compressive deformations occur *in vivo* in cartilage and with the previous demonstrations of several *in vitro* chondrocyte responses to both types of loading, it is of interest to compare different responses between shear and compression. While many prior studies have investigated compressive loading and its effects on cellular mechanotransduction, few studies have examined the role of metabolism. Further, no study to data has investigated how chondrocytes respond metabolically to both shear and compressive loading. Therefore, the objective of this study is to identify mechanosensitive differences between shear and compressive stimulation after 0, 15, and 30 min of 5±1.9% cyclical strain at 1.1 Hz for SW1353 chondrocytes, encapsulated in physiologically stiff agarose. After loading, metabolite extracts were analyzed by liquid chromatography-mass spectrometry (LC-MS) in search of metabolites that differentiate between the type and duration of loading. These data provide insight into how SW1353 chondrocytes respond to mechanical stimuli thus expanding our knowledge of chondrocyte mechanotransduction.

## RESULTS

**Fig. 1.**
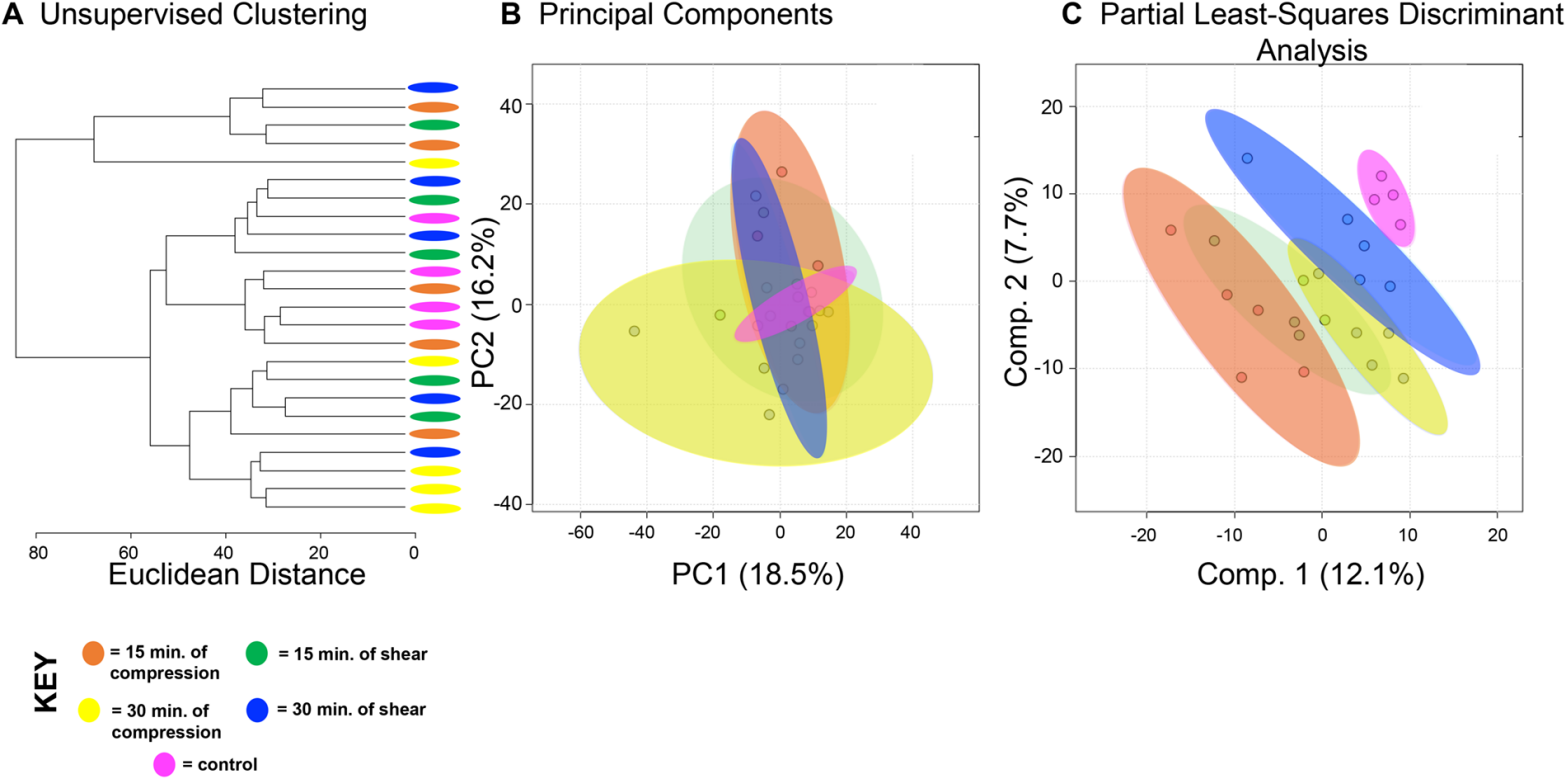
**SW1353 chondrocytes have shared and distinct responses to shear and compressive forces.** A total of 1457 metabolites were analyzed by both unsupervised hierarchical clustering (HCA) and principal component analysis (PCA) and supervised partial least-squares discriminant analysis (PLS-DA). (A) Unsupervised HCA visualized by a dendrogram did not distinguish distinct clusters between the five experimental groups (control, 15 min compression and shear, 30 min compression and shear). HCA assigns Euclidean distances to illustrate dissimilarity between samples and reveal clusters of samples with similar metabolomic profiles. (B) PCA, like HCA, did not display distinguished clusters between the five experimental groups. PCA is shown as a scatterplot with the first two PC on the x and y axes. The x axis shows PC1, which accounts for 18.3% of the variation in the dataset. PC2 is on the y axis and accounts for 14.2% of the variation in the dataset. (C) Supervised PLS-DA finds some separation between the five experimental groups, with similarity between different time points and different forces. PLS-DA is shown as a scatterplot of the top two components, with component 1 accounting for 14.6% and component 2 accounting for 7.6% of the variation in the dataset. The colors in A–C correspond to sample cohorts: pink, control; orange, 15 min compression; yellow, 30 min compression; green, 15 min shear; blue, 30 min shear. *n*=5 samples per group.

A total of 1457 distinct metabolite features were detected across all experimental groups (Table S1). First, all experimental groups (control, 15 min compression and shear, 30 min compression and shear) were analyzed (Fig. 1). Hierarchical cluster analysis (HCA) and principal component analysis (PCA) examined overall variation between groups in the dataset. Neither shows complete separation between experimental groups (Fig. 1A,B), although the first two principal components are associated with more than 30% of the overall variance indicating moderate underlying structure in the dataset. To further examine this dataset, we applied PLS-DA to assess differences between experimental groups. Separation between experimental groups was observed with minimal overlap (Fig. 1C) using this supervised method.

**Table 1.**
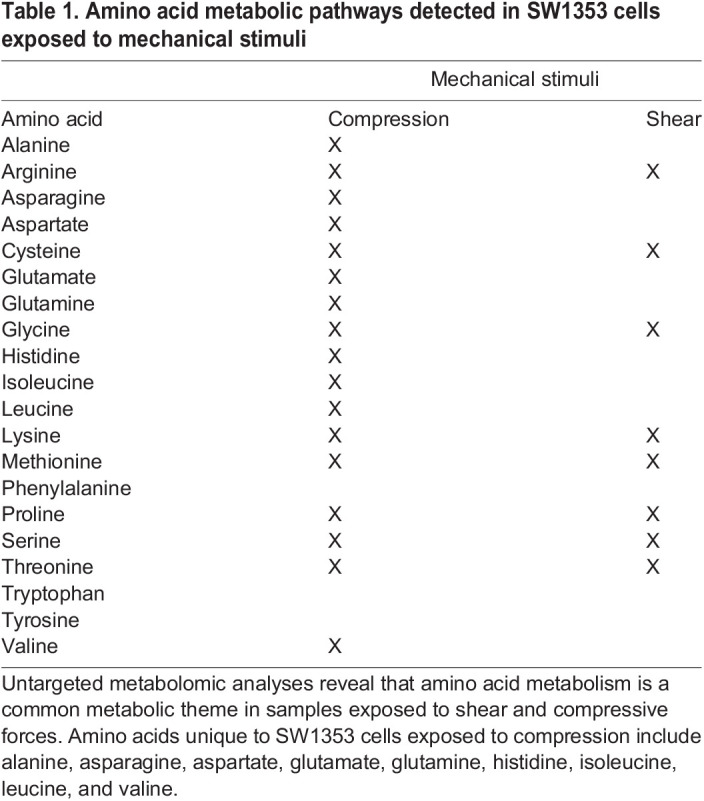
Amino acid metabolic pathways detected in SW1353 cells exposed to mechanical stimuli

**Fig. 2.**
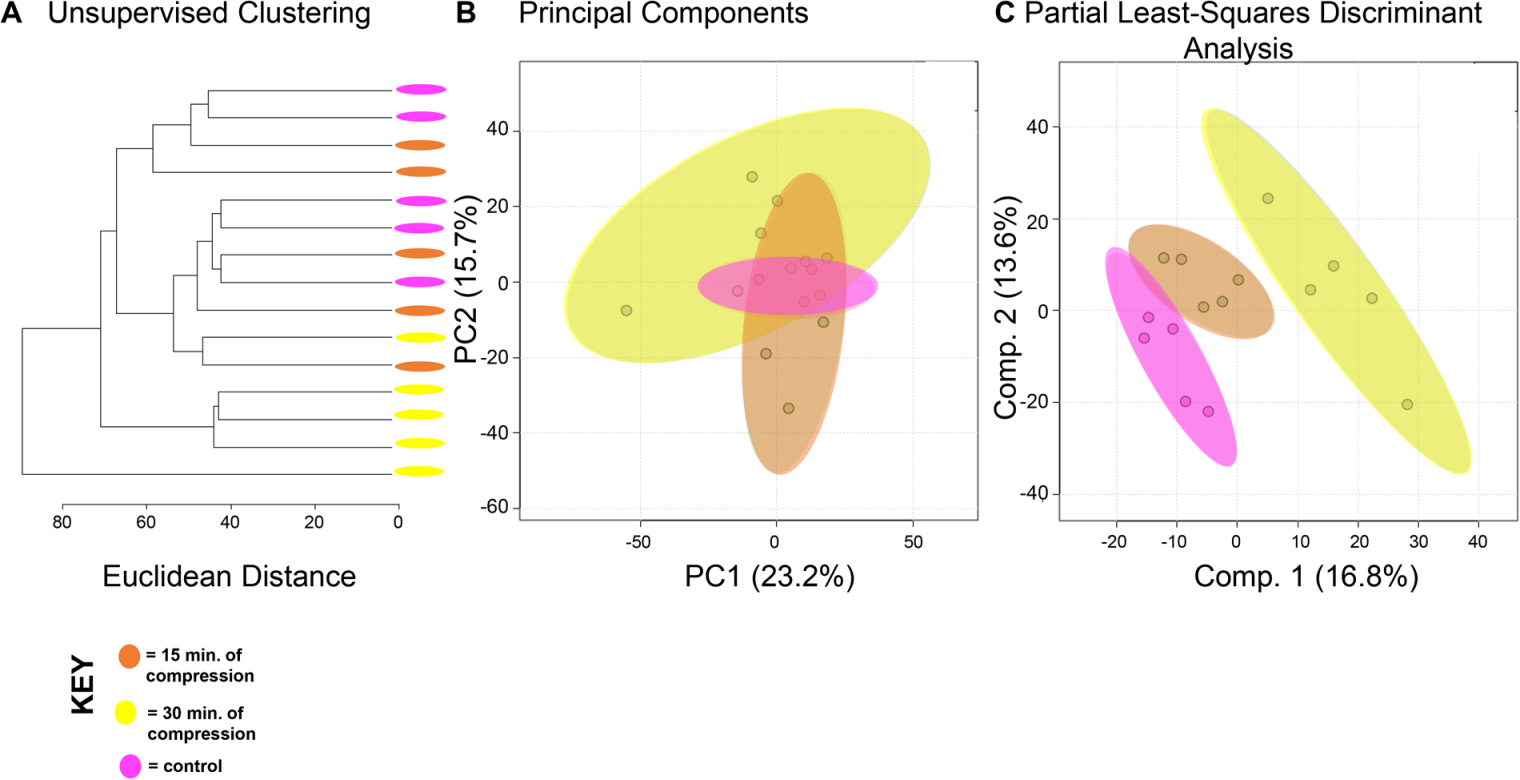
**Differing amounts of exposure to compression reveal distinct metabolic profiles.** Comparison of samples exposed to compression reveal metabolomic profiles of chondrocytes between 15 and 30 min of compression differ. (A) Samples compressed at different times separate into distinct clusters by HCA as illustrated in the dendrogram. (B) PCA displays some clustering of samples within their respective cohorts: chondrocytes exposed to compressive forces for 15 min (orange) and 30 min (yellow). Control chondrocytes that were not exposed to mechanical stimuli are displayed for comparison purposes (pink). PCA is shown as a scatterplot of the first two PCs (PC1 and PC2), which account for 23.2% and 15.7% of the overall variation in the dataset, respectively. (C) PLS-DA finds clear separation between samples. PLS-DA is visualized as a scatterplot of the top two components, which account for 16.8% and 13.6% of the overall variation in the dataset. *n*=5 samples per group.

**Fig. 3.**
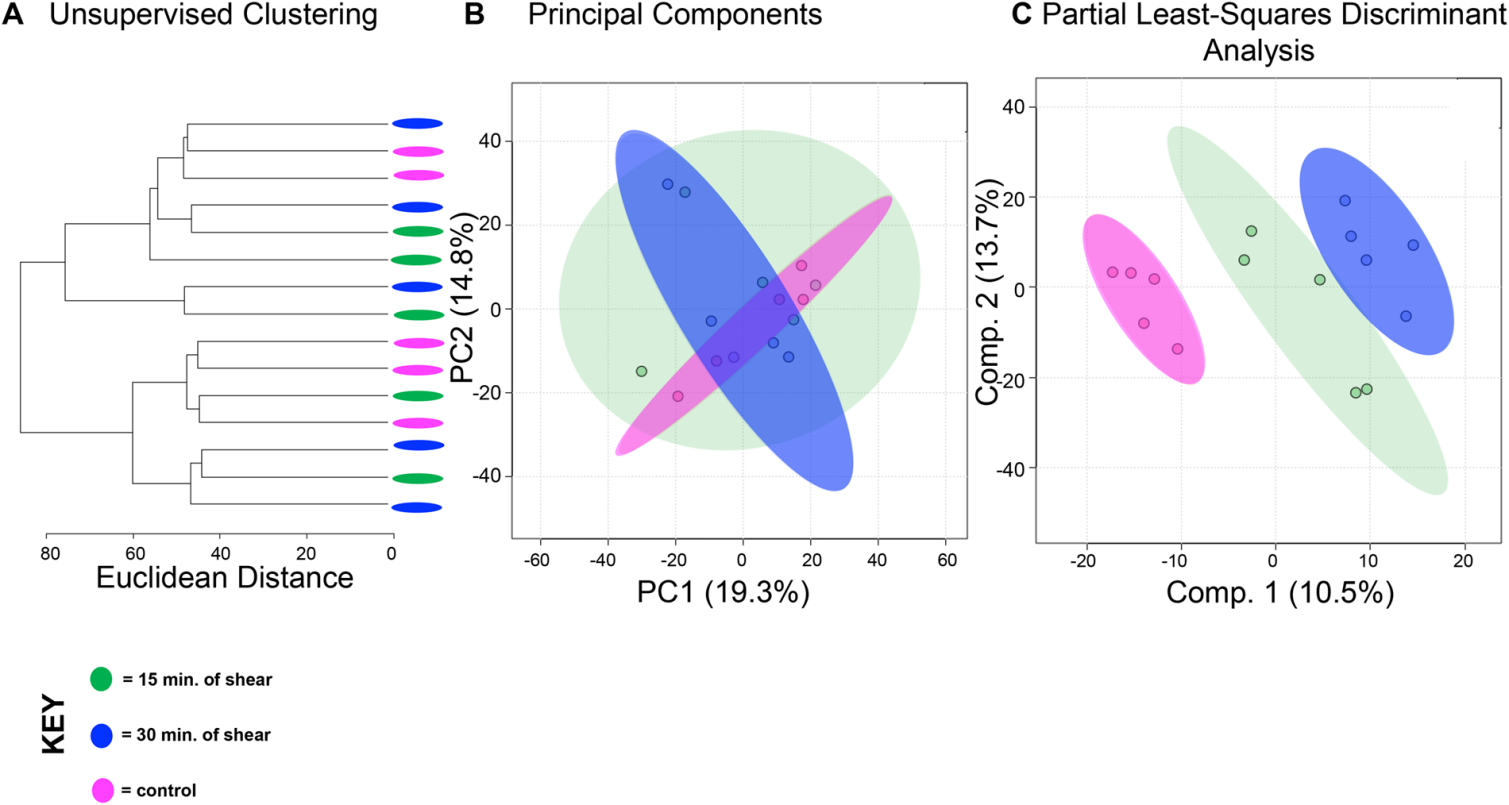
**Differing amounts of shear force reveal distinct metabolic profiles.** Metabolomic profiles of chondrocytes exposed to 15 and 30 min of shear force differ. (A) Samples compressed at different times separate into weakly distinct clusters by HCA as illustrated in the dendrogram. (B) PCA displays some clustering of samples within their respective cohorts: chondrocytes exposed to shear force for 15 min (green) and 30 min (blue). Control chondrocytes that were not exposed to mechanical stimuli is displayed for comparison purposes (pink). PCA is shown as a scatterplot of the first two PCs (PC1 and PC2), which account for 19.3% and 14.8% of the overall variation in the dataset, respectively. (C) PLS-DA finds clear separation between samples. PLS-DA is visualized as a scatterplot of the top two components, which account for 10.5% and 13.7% of the overall variation in the dataset. *n*=5 samples per group.

To compare the effects of shear and compression on metabolomic profiles, samples exposed to each loading type, shear and compression, were analyzed separately (Figs 2,3). When analyzing differences between compressive samples between 0, 15, and 30 min of loading, HCA and PCA find clustering of samples within their respective time points indicating chondrocyte mechanotransduction (Fig. 2A,B). Partial least-squares discriminant analysis (PLS-DA) shows further discrimination with all samples clustering within their respective cohorts (Fig. 2C). We found similar results for shear stimulation. HCA and PCA revealed clustering of samples within each time point (Fig. 3A,B). PLS-DA shows further separation between samples from different shear groups (Fig. 3C). These data show that SW1353 chondrocytes respond to both shear and compression with alterations in their metabolomic profiles.

**Fig. 4.**
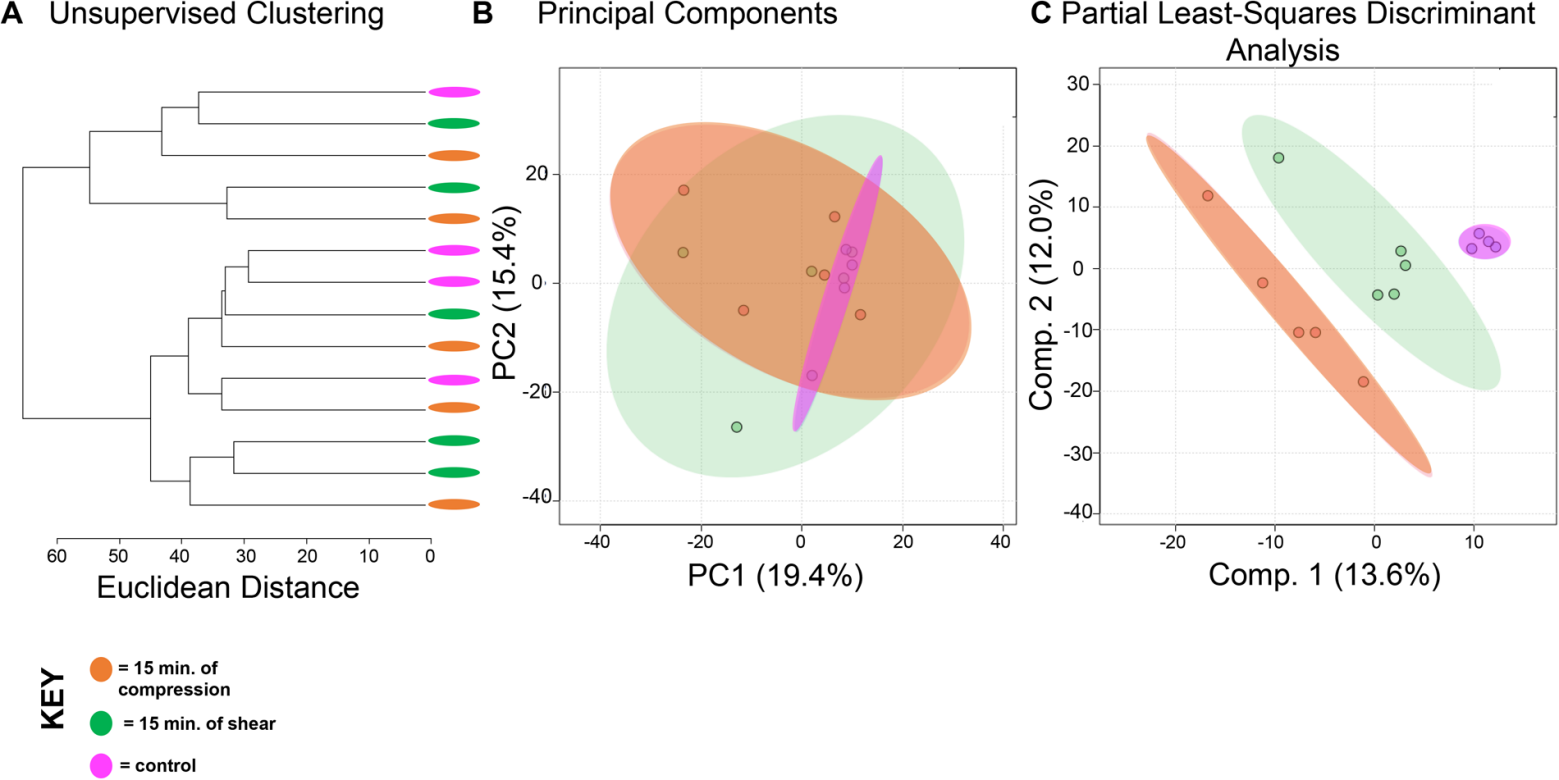
**Metabolic profiles of differing forces with an equal exposure time of 15 min vary.** Metabolomic profiles of chondrocytes exposed to compression and shear forces for 15 min differ metabolically. (A) Samples exposed to different forces for the same amount of time somewhat separate into clusters by HCA as illustrated in the dendrogram. (B) PCA displays some clustering of samples within their respective cohorts: chondrocytes exposed to compression for 15 min (orange) and chondrocytes exposed to shear forces for 15 min (green). Control chondrocytes that were not exposed to mechanical stimuli is displayed for comparison purposes (pink). PCA is shown as a scatterplot of the first two PCs (PC1 and PC2), which account for 19.4% and 15.4% of the overall variation in the dataset, respectively. (C) PLS-DA finds clear separation between samples. PLS-DA is visualized as a scatterplot of the top two components, which account for 13.6% and 12.0% of the overall variation in the dataset. *n*=5 samples per group.

**Fig. 5.**
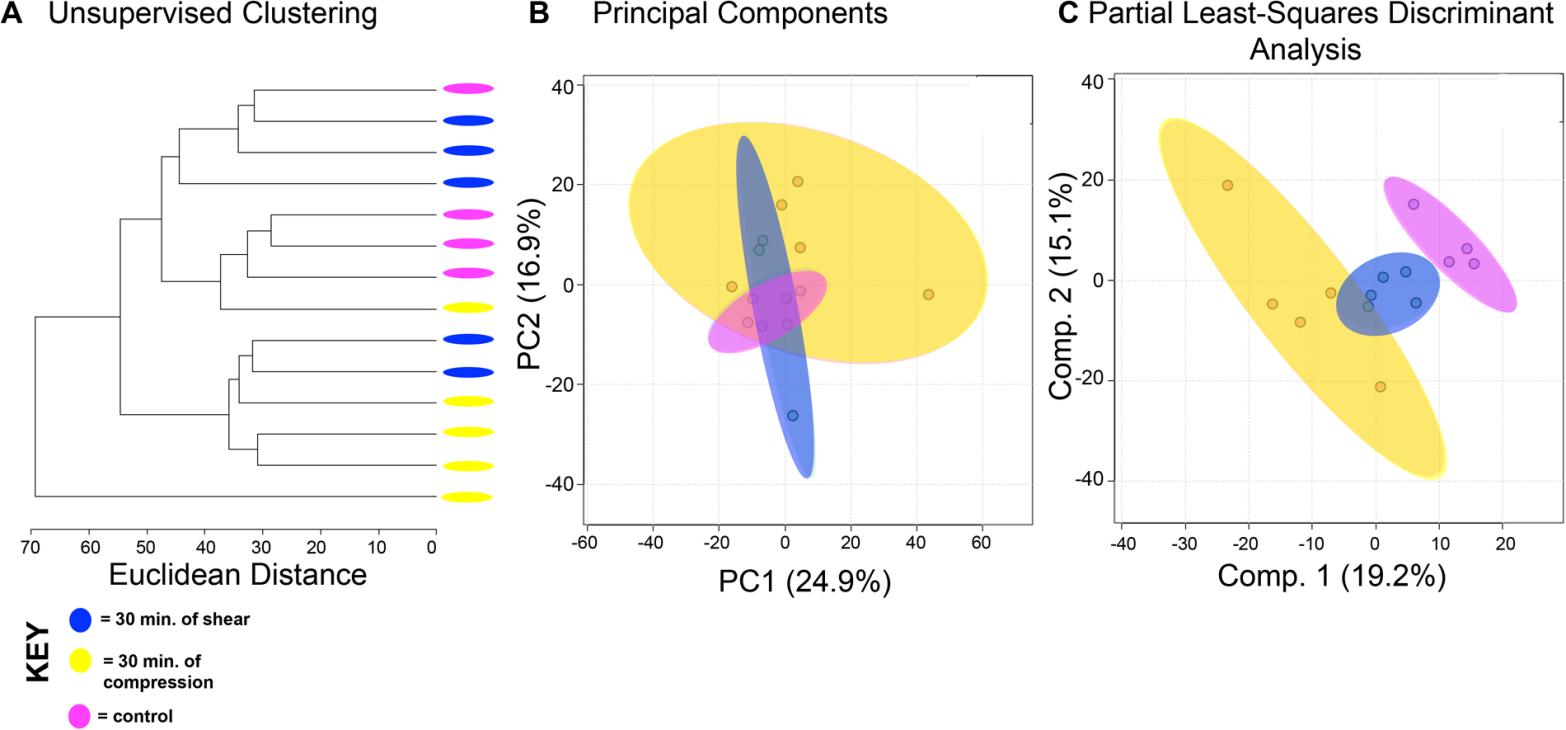
**Metabolic profiles of differing forces with an equal exposure time of 30 min vary.** Metabolomic profiles of chondrocytes exposed to compression and shear forces for 30 min display distinct metabolomic profiles. (A) Samples exposed to different forces for the same amount of time somewhat separate into clusters by HCA as illustrated in the dendrogram. (B) PCA displays clear clustering of samples within their respective cohorts: chondrocytes exposed to compression for 30 min (yellow) and chondrocytes exposed to shear forces for 30 min (blue). Control chondrocytes that were not exposed to mechanical stimuli is displayed for comparison purposes (pink). PCA is shown as a scatterplot of the first two PCs (PC1 and PC2), which account for 24.9% and 16.9% of the overall variation in the dataset, respectively. (C) PLS-DA finds clear separation between samples. PLS-DA is visualized as a scatterplot of the top two components, which account for 19.2% and 15.1% of the overall variation in the dataset. *n*=5 samples per group.

Chondrocytes exposed to shear and compression for the same amount of time showed interesting similarities and differences between types of mechanical stimulation. When comparing compression and shear at 15 min, HCA and PCA showed limited separation and clustering (Fig. 4A,B). But PLS-DA clearly distinguishes samples between shear and compression at the 15-min time point (Fig. 4C). Similarly, compression and shear samples that were both exposed to 30 min of stimulation were compared. Both HCA and PCA found clear separation and clustering of samples at 30 min (Fig. 5A,B) that was confirmed by PLS-DA (Fig. 5C). With this knowledge, we used VIP scores from PLS-DA and volcano plot analysis to identify specific metabolites that contributed to the differences between experimental groups. Specific metabolite features of interest were then matched to metabolite identities and pathways using MetaboAnalyst. Significant pathways consist of sialic acid (SA) metabolism (all experimental groups); prostaglandin formation from arachidonate, fatty acid metabolism, tyrosine metabolism (15 min of compression versus 30 min of compression); porphyrin metabolism (15 min of shear versus 30 min of shear); carnitine shuttle (15C versus 15S); squalene and cholesterol biosynthesis (30C versus 30S).

**Fig. 6.**
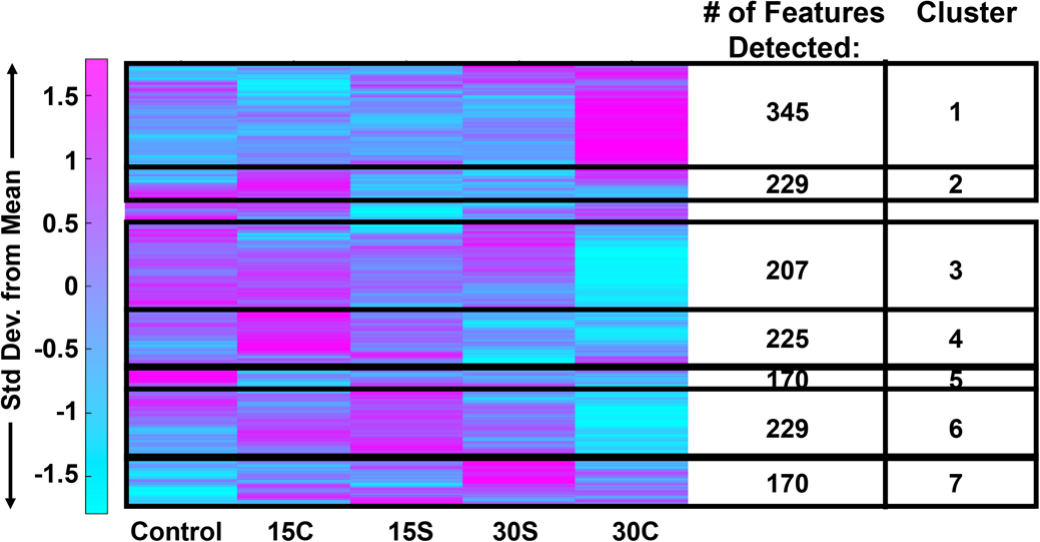
**Heatmap analysis reveals that metabolic phenotypes differ when exposure time and mechanical force differ.** Median intensities were clustered into five groups: control samples (*n*=4), 15 min of compression (*n*=5), 30 min of compression (*n*=5), 15 min of shear (*n*=5) and 30 min of shear force (*n*=5). 1457 metabolite intensities were detected in SW1353 cells. To determine associated pathways and metabolites, *MS Peaks to Pathways* was employed. Median values of *n*=5 samples per group.

Co-regulated metabolite features and associated pathways between shear and compressive stimulation were identified using heatmap analysis. Using the median metabolite intensities and HCA, seven clusters were identified based on Euclidian distance. Metabolites from these clusters were then used to determine associated pathways (Fig. 6). In total, 372 pathways were detected, and 80 of the 372 are statistically significant (*P*<0.05).

Cluster 1 contained 354 metabolite features that were upregulated in samples exposed to 30 min of compression. These mapped to 25 statistically significant enriched pathways including primary bile acid biosynthesis, histidine metabolism, fructose and mannose metabolism, pyrimidine and purine metabolism, and alanine, aspartate, and glutamine metabolism. Cluster 2 and 4 contained 229 and 225 metabolite features, respectively, which were upregulated in samples exposed to 15 min of compression. These features corresponded to 23 statistically significant enriched pathways including various amino acid metabolic pathways.

Cluster 3 contained 207 metabolite features that were downregulated in samples exposed to 30 min of compression. These mapped to seven statistically significant enriched pathways including alanine, aspartate, and glutamate metabolism, *N*-glycan biosynthesis, mannose type O-glycan biosynthesis, arginine biosynthesis, steroid hormone biosynthesis, and pyrimidine metabolism.

Cluster 5 containing 170 metabolite features that were upregulated in control samples. These features corresponded to eight statistically significant enriched pathways including butanoate metabolism, sphingolipid metabolism, propanoate metabolism, the citrate cycle (TCA cycle), histidine metabolism, and various amino acid metabolic pathways.

Clusters 6 and 7 contained 229 and 170 metabolites that corresponded to samples exposed to shear for both 15 and 30 min. The features of the two combined clusters corresponded to 17 statistically significant enriched pathways including various amino acid metabolic pathways, the pentose phosphate pathway, glycolysis and gluconeogenesis.

Amino acid metabolic pathways unique to compressed SW1353 cells include alanine, asparagine, aspartate, glutamate, glutamine, histidine, isoleucine, leucine, and valine metabolism. Metabolic pathways shared by both SW1353 cells exposed to compression and shear included arginine, cysteine, glycine, lysine, methionine, proline, serine and threonine. Amino acids that were not detected within this dataset include phenylalanine, tryptophan, and tyrosine. All significant (*P*<0.05) detected pathways were investigated and compared to findings in the literature. In the Discussion, we focus on pathways already established in chondrocyte mechanotransduction. All pathway results are found in the Supplementary Material. Those that were detected in this study but have yet been detected nor discussed by others were not examined further (Table S2).

## DISCUSSION

Cartilage provides a smooth surface for movement, reduces stress placed on underlying bone, and requires a strong and smooth ECM to resist high loads created in the joint. To further investigate the role of cartilage and mechanotransduction in chondrocytes, we investigated the effects of mechanical stimuli on the ECM and how differing types of load (shear and compression) and exposure times affect the metabolism of SW1353 chondrocytes. In total, 1457 metabolites were detected, and metabolic profiles were generated for each experimental condition (time point 0, 15, 30 min, mechanical stimulus – shear or compression).

In every pairwise comparison, metabolomic profiles from different experimental groups differed from each other. When comparing all experimental groups, SA metabolism was the most notable pathway determined from the top 100 VIP metabolites when comparing all groups. This finding corresponds to SA being an inflammatory biomarker for OA and rheumatoid arthritis (RA), while also contributing to the lubrication of the joint (Alturfan et al., 2007). Additionally, lipid metabolism (fatty acid metabolism, cholesterol biosynthesis, prostaglandin formation from arachidonate) was upregulated in compressed samples. Based on potential crosstalk between joint tissues, the permeability of AC, and the ability of fats like phospholipids to act as lubricants, overexpression of fat associated pathways may lead to cartilage lesions, joint space narrowing, joint immobilization, and ultimately contributing to the development of OA (Appleton et al., 2007; Lotke and Granda, 1972; Maroudas et al., 1968; Pan et al., 2012; Sohn et al., 2012; Villalvilla et al., 2013). Central energy metabolism and beta-oxidation were present across experimental groups and strongly upregulated amongst compressed samples (TCA cycle, carnitine shuttle). We interpret this as exposure to mechanical stimuli driving a high chondrocyte ATP demand: the TCA cycle and the carnitine shuttle may have been upregulated to meet this need. Amino acid metabolism differed between samples exposed to shear and compression while several specific amino acids were unique to compression. Taken together, compression may induce mechanosensitive pathways that are needed to produce a broader set of products than shear such as amino acids, both non-essential and essential.

Many studies have shown the diverse effects of mechanical stimulation on the musculoskeletal system, chondrocytes, ECM, cell metabolism, and its relation to OA (Jutila et al., 2014, 2015; Salinas et al., 2017; Zignego et al., 2015). Bushmann et al. investigated the effects of static and dynamic mechanical compression on matrix biosynthesis, chondrocyte proliferation, and quantified proteoglycan and glycosaminoglycan content. Using cell culture and radiolabeling, *in vitro* chondrocytes form a mechanically functional matrix that preserves certain physiological features of chondrocyte behavior and response to physical stimuli (Buschmann et al., 1995, 1992). O'Connor et al. examined the effects of mechanical stimulation on chondrocyte metabolism and transient receptor potential vanilloid 4 (TRPV4). The results of this study suggest that dynamic loading has a profound effect on cell physiology, ion channel function, and TRPV4 mediated mechanotransduction (O'Conor et al., 2014).

Jutila et al. report the effects of compressive loading at early time points (0, 15 and 30 min). Through the utilization of targeted and untargeted metabolomics, 54 metabolites were found to mediate chondrocyte mechanotransduction (Jutila et al., 2014, 2015). Other studies identified the effects of physiological compression on energy metabolism and maintenance of the pericellular and extracellular matrices (Salinas et al., 2017; Zignego et al., 2015). In addition, many pathways related to the metabolism of energy, lipids, and amino acids were identified through targeted and untargeted metabolomics. The results of our study are consistent with these prior studies and together indicate that extended exposure to mechanical stimuli affects chondrocytes, ECM components, and alters cellular metabolism.

### Inflammation

Exposure to shear and compression lead to metabolic shifts associated with inflammation and deterioration in SW1353 cells. The 100 most significant metabolites across all sample groups were involved in SA metabolism (Fig. 1). SA is an acylated derivative of neuraminic acid attached to glycoproteins and glycolipids. Serum SA levels are a known marker of inflammation and has been reported as a useful biomarker of inflammation in patients with OA and RA (Cui et al., 2014). Additionally, studies find that differing levels of SA are linked to OA severity and could be used as a diagnostic tool in the future (Alturfan et al., 2007; Browning et al., 2004; Chavan et al., 2005; Cui et al., 2014; Gobezie et al., 2007; Kosakai, 1991). Our study provides further evidence that SA production could be a potential marker for OA due to its metabolic presence when mechanically stimulated with shear and compressive forces. SA is also a key component of the superficial zone protein lubricin that decreases cartilage-on-cartilage friction (Carlson et al., 2019; Jay et al., 2012; Jutila et al., 2014, 2015). Therefore, these findings may suggest different loading types and exposure times may promote changes in metabolism that correspond to changes in cartilage structure and function. Taken together, this is the first study to find SA metabolism to be upregulated after mechanical stimuli and further research may identify its effects on the ECM, cartilage health, and OA.

Further analysis showed that prostaglandin formation from arachidonate was a significant pathway associated with compression. Prostaglandin metabolites were upregulated after 30 min of compression. Studies show that prostaglandin formation from arachidonate can be triggered by obesity, age, and mechanical stress (Bar-Or et al., 2015). Individually or combined, these stimuli result in cartilage deterioration which can be attributed to the innate immune system, by pathogen-associated molecular patterns (PAMPs), damage-associated molecular patterns (DAMPs), and alarmins (Bianchi, 2007). Alarmins are triggered by signals from cellular damage, abnormal proteins, leaky vasculature, and fragments of cartilage matrix (Bar-Or et al., 2015; Bianchi, 2007). Prostaglandins such as PGD2, PGE2, and others are anti-inflammatory molecules that initiate a decrease in inflammation and trigger recovery to normal cellular function (Attur et al., 2008; Wang et al., 2013, 2010). Upon the application of low fluid shear stress anti-inflammatory molecules are activated, inflammation is halted, and recovery is initiated (Attur et al., 2008; Bar-Or et al., 2015; Wang et al., 2013). The results of this study suggest that increased compressive loading leads to an increased presence of inflammatory pathways such as prostaglandin formation from arachidonate in SW1353 cells. Therefore, if confirmed in primary cells, increased compressive stimulation may result in increased inflammation, which in excess could lead to OA if not balanced by other physiological processes.

### Lipid metabolism

In this study, a common metabolic theme of SW1353 cells stimulated by compression was upregulation of metabolite features corresponding to lipid metabolism. The pathways detected include fatty acid biosynthesis, cholesterol biosynthesis, and prostaglandin formation from arachidonate which were significantly upregulated after 30 min of compression. Further, butanoate and sphingolipid metabolism were significantly upregulated in control samples, implying downregulation upon mechanical stimulation.

When joint homeostasis is altered, increased protease activity can result in cartilage lesions, joint space narrowing, and ultimately, breakdown of the tissues (Appleton et al., 2007; Lotke and Granda, 1972; Maroudas et al., 1968; Pan et al., 2012; Sohn et al., 2012; Villalvilla et al., 2013). Hence cartilage health and metabolism greatly rely on the chondrocyte microenvironment. On the metabolic level, both fatty acid metabolism and cholesterol biosynthesis are required to maintain healthy ECM and cartilage (Aguilar et al., 2009; Bernstein et al., 2010; Gkretsi et al., 2011; Wu and De Luca, 2004). Cartilage contains stores of lipid deposits, especially in chondrocytes, during times of health and disease. Expected lipid content in both healthy and pathological cartilage include palmitic, linolic, and oleic acid, but fatty acids and arachidonate acid are elevated in OA samples and associated with increased histological severity (Lippiello et al., 1991). Beyond prostaglandin synthesis from arachidonate, in this study both fatty acid metabolism and cholesterol biosynthesis were upregulated in loaded samples indicating that chondrocyte metabolism was altered as a result of shear and compression stimulation. Future studies may determine the role of mechanical stimuli in regulating the cartilage ECM through these pathways.

### Central metabolism

Injurious mechanical stimuli, traumatic injury, and other factors such as obesity, age, and gender initiate the breakdown of cartilage leading to OA. In contrast, lower levels of mechanical stimuli result in matrix synthesis to support cartilage homeostasis. Both repair and maintenance require additional energy production to produce ATP. Pathways that generate ATP are pathways related to central metabolism including glycolysis, the TCA cycle, beta-oxidation, and the carnitine shuttle. In this study, pathways related to the glycolysis, TCA cycle, and the carnitine shuttle were significantly upregulated consistent with prior results (Salinas et al., 2017; Salinas et al., 2019; Zignego et al., 2015).

The carnitine shuttle was upregulated in all mechanically stimulated samples after 15 min of either compression or shear. Beta-oxidation breaks down fatty acids to produce energy and is regulated by the carnitine shuttle. Carnitines stimulate cell proliferation, induce ATP synthesis, and serum carnitines were associated with OA grade (Tootsi et al., 2018; Zhai, 2019).

Metabolites and corresponding pathways involved in glycolysis and the TCA cycle were upregulated in SW1353 cells exposed to 15 min of shear. These pathways were the only two detected pathways unique to shear force. In chondrocytes, anaerobic glycolysis is restricted as oxygen and nutrients are limited in avascular cartilage. But previous studies have found that both aerobic and anaerobic glycolysis occur in chondrocytes (Zheng et al., 2021). Specific to OA, the rate of anerobic glycolysis in diseased cartilage is higher compared to healthy cartilage (Maneiro et al., 2003). Other intermediates that can be incorporated into glycolysis to yield energy are fructose, mannose, and galactose. In this study, fructose, mannose, and galactose metabolism were upregulated in SW1353 cells exposed to compression.

The TCA cycle produces ATP from pyruvate and other sources such as sugar, fat, and proteins. Intermediate TCA metabolites include citrate, malate, succinate, fumarate, and various others. Studies find that patients with OA have higher levels of synovium metabolites associated with the TCA cycle compared to healthy controls (Adams et al., 2012; Anderson et al., 2018; Zhai, 2019). The results of this study provide evidence that mechanical stimulation by compression specifically generates additional ATP. This ATP may be used to maintain and produce matrix thus requiring upregulation of the TCA cycle and the carnitine shuttle.

Beyond glycolysis and the TCA cycle, the pentose phosphate pathway (PPP) was upregulated in SW1353 cells exposed to compression. The PPP pathway utilizes glucose-6-phosphate, which is a product of glycolysis. The PPP gives rise to NADH and ribose-5-phosphate which is used to make subunits for DNA, RNA, and amino acids such as histidine from phosphoribosyl pyrophosphate (PRPP). Here, histidine and beta-alanine metabolism were upregulated only in SW1353 cells exposed to compression. Histamine is the decarboxylated amine form of histidine, and previous studies have found that histamines stimulate chondrocytes proliferation in humans (Tetlow and Woolley, 2003, 2005). Therefore, the upregulation of histidine metabolism in this study suggests that SW1353 cells exposed to compression may proliferate in response mechanical stimuli.

Finally, two pathways detected in SW1353 cells stimulated by 30 min of compression were pyrimidine and purine metabolism (*P*<0.05). Pyrimidine and purine metabolism stem from the pentose phosphate pathway. These two pathways function to recycle nucleosides and participate in *de novo* nucleotide synthesis. Upregulation of these pathways may relate to an increase in cellular demand of complex molecules such as RNA upon mechanical stimulation (e.g. to support transcription). Beyond nucleotide and nucleoside production, pyrimidine and purine metabolism give rise to various amino acids. In particular, pyrimidine interconversions can lead to beta-alanine metabolism. Previous studies have found increased levels of beta-alanine in urine, synovium, synovial fluid, and in subchondral bone in OA animal models (Hugle et al., 2012; Maher et al., 2012; Okun et al., 2002; Yang et al., 2016).

### Amino acid metabolism

**Fig. 7.**
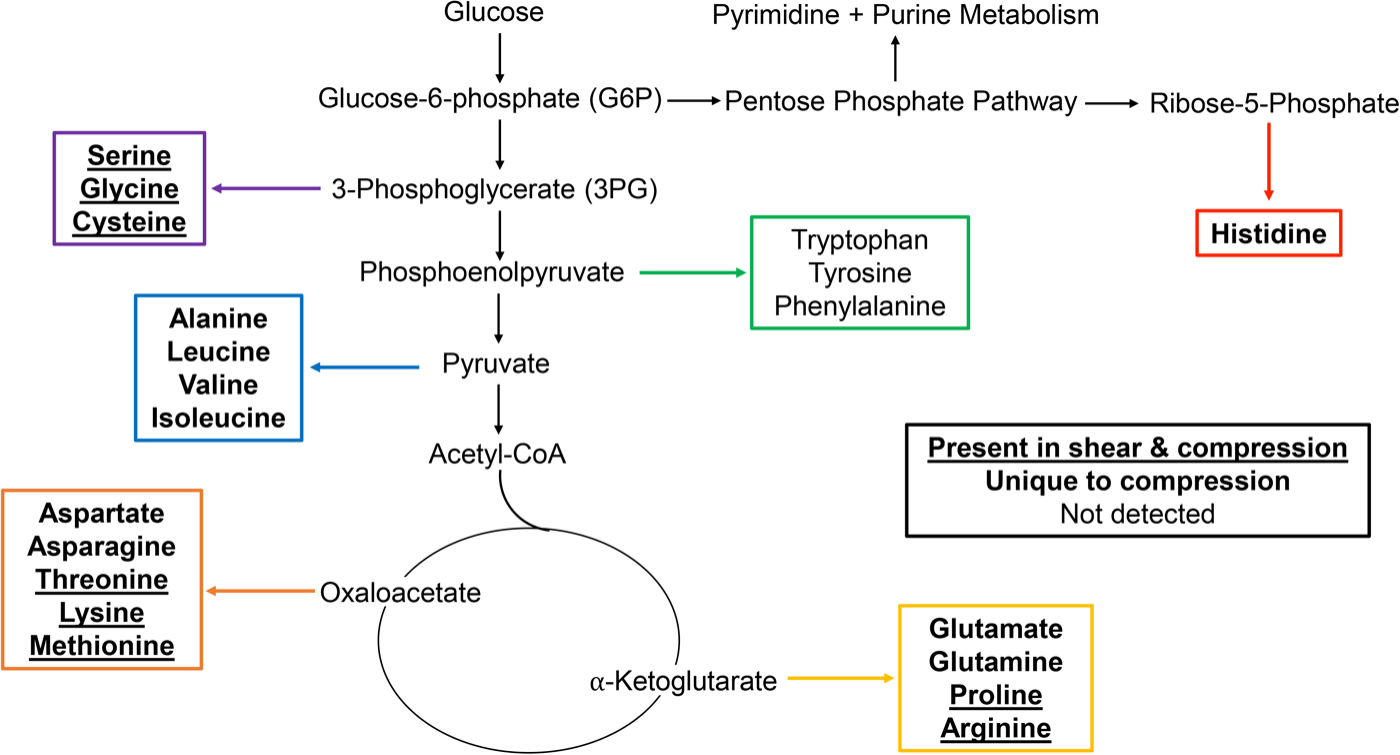
**Mapping of mechanically stimulated amino acids in metabolic pathways.** Amino acids that are bolded and underlined were upregulated in SW1353 cells that were exposed to compression and shear force. Those bolded only were upregulated in cells exposed to compression. Amino acids that are not bolded nor underlined were not detected (tryptophan, tyrosine, phenylalanine). Intermediates of interest include ribose-5-phosphate, 3-phosphoglycerate, pyruvate, α-ketoglutarate and oxaloacetate. Additionally, pyrimidine metabolism was upregulated in samples exposed to 30 min of compression that stems from the pentose phosphate pathway.

Metabolomic profiles for SW1353 cells stimulated by either shear or compression show that amino acid metabolism is commonly upregulated by these types of loading with certain responses specific to the type and/or duration of loading. Amino acid pathways unique to compressive stimulation include metabolism of alanine, asparagine, aspartate, glutamate, glutamine, histidine, isoleucine, leucine, and valine metabolism. In contrast, arginine, cysteine, glycine, lysine, methionine, proline, serine, and threonine were induced by both shear and stimuli compression (Fig. 7).

Amino acids are the building blocks of proteins and determine structure and function. Depending on cellular demand, amino acids can be converted into intermediates, or produced from glucose, through central energy metabolism. The results of this study suggest that major precursors of interest when analyzing the relationship between mechanical stimuli and metabolism include ribose-5-phosphate (histidine), 3-phosphoglycerate (serine, glycine, cysteine), pyruvate (alanine, leucine, valine, isoleucine), α-ketoglutarate (glutamate, glutamine, proline, arginine) and oxaloacetate (aspartate, asparagine, threonine, lysine, methionine) (Fig. 7). The results of this study suggest that the upregulation of metabolic pathways may correspond to the synthesis of essential and non-essential amino acids that are needed to synthesize musculoskeletal associated proteins in response to mechanical stimuli. Furthermore, the application of different stimuli, such as compression and shear, results in different metabolic profiles that impact synthesis of various proteins.

These data show that there are clearly differences between the metabolomic profiles of SW1353 chondrocytes exposed to shear and compression. These differences may correlate to the differences between the mechanical stimuli. This agarose system appears isotropic in 2D analyses with shear strains ∼10% smaller than compressive strains for uniaxial loading (Zignego et al., 2014). Yet, chondrocyte mechanotransduction is likely to include shear-specific, compression-specific, and shared responses. While the untargeted approach used here found candidate pathways, a targeted approach in future studies is required to determine if a particular response is specific to either shear or compression.

### Limitations

This study has important limitations and opportunities for future studies. First, the behavior of the chondrocyte cell line SW1353 cells may differ from primary human chondrocytes. Second, although our *in vitro* approach is required to apply well-defined mechanical loads, this approach may yield different results from an *in vivo* model or one where the pericellular matrix is present. Third, exposure to mechanical stimuli for both periods shorter than 15 min and beyond 30 min could expand our knowledge on the varying metabolic phenotypes beyond the those in this study. Finally, this was an untargeted metabolomic study. By performing a more targeted metabolomic analysis utilizing MS/MS, uncertainty of predicted identities of key metabolite features and their associated pathways can be reduced.

## Conclusion

To our knowledge, this is the first study to generate global metabolomic profiles of SW1353 cells exposed to both shear and compression at early time points. Pathways were identified within experimental groups, which provide insight into the role of the extracellular matrix, articular cartilage homeostasis, and the potential outcomes of metabolic alterations that ultimately lead to osteoarthritis. Furthermore, these metabolomic profiles support the link between mechanical stimuli and cartilage remodeling. Expansion of this study may identify cartilage loading protocols to bolster matrix synthesis that may be relevant to drug development.

## MATERIALS AND METHODS

### SW 1353 chondrocyte culture and encapsulation

SW1353 chondrocytes (gift of M. Lotz, The Scripps Research Institute, La Jolla, CA, USA) were cultured in DMEM with 10% fetal bovine serum and antibiotics (10,000 I.U./mL penicillin and 10,000 μg/ml streptomycin). Cells were first expanded in monolayer for one passage at 5% CO_2_ at 37°C prior to gel encapsulation. For encapsulation, cells were seeded in physiologically stiff agarose (4.5% v/v, Sigma-Aldrich; Type VII-A A0701) at a concentration of 500,000 cells/hydrogel (diameter: 7 mm, height: 12.7 mm). Hydrogel constructs were individually placed in wells, submerged in DMEM with 10% fetal bovine serum, and allowed to equilibrate for 24 h prior to mechanical stimulation. A sample size of *n*=5 was selected for this initial experiment as prior data were not available for a power analysis.

### Mechanical stimulation

Cell encapsulated hydrogels were randomly assigned to five experimental groups (*n*=5 per experimental group): unloaded controls (0 min of mechanical stimuli), 15 or 30 min of cyclical shear, or 15 or 30 min of cyclical compression. Hydrogels were placed in DMEM with 10% fetal bovine serum without antibiotics and mechanically stimulated with cyclical shear or compression using a custom-built sinusoidal loading apparatus. Sinusoidal compressive and shear strains of 5.00±1.90% (based on initial 12.70 mm±0.01 mm gel height and 7.00 mm±0.01 mm gel diameter, respectively) were applied at 1.1 Hz to simulate the preferred stride rate of humans for 0, 15 and 30 min (Jutila et al., 2014, 2015). All hydrogel mechanical testing was performed in physiological cell culture conditions (5% CO_2_, 37°C).

### Metabolite extractions

Immediately after mechanical stimulation, hydrogels were flash frozen in liquid nitrogen, pulverized and placed in 70:30 (v/v) methanol:acetone. Samples were vortexed for 5 min and placed in −20°C for 5 min. This two-step process was repeated four times and samples were stored overnight (−20°C) for macromolecule precipitation. The next day proteins and other macromolecules were pelleted by centrifugation. The supernatant containing the metabolites was transferred to a separate tube and dried by vacuum concentration for 6.5 h to remove solvents. Dried metabolites were resuspended in 100 µl mass spectrometry grade 50:50 (v/v) water:acetonitrile solution immediately prior to high performance liquid chromatography-mass spectrometry (HPLC-MS) analysis.

### Untargeted metabolomic analysis

Metabolomics is the analysis of small molecules (metabolites) in a biological system that provides a global description of cellular function at a given point in time. Extracted metabolites were analyzed using HPLC-MS (Agilient 6538 Q-TOF mass spectrometer) in positive mode (resolution: ∼20 ppm, accuracy: ∼5 ppm, possible ionization adducts: H^+^, Na^+^). Peak intensities for m/z values in the experimental sample set were identified and exported using Agilient MassHunter Qualitative Analysis software. All data was log transformed and autoscaled prior to analysis. All statistical analyses utilized the transformed and scaled data using MetaboAnalyst (Xia and Wishart, 2016).

Univariate, supervised, and unsupervised multivariate analyses were used to visualize and narrow the dataset. The unsupervised multivariate statistical analyses that were utilized were HCA and PCA. HCA is used to visualize metabolomic profiles, identify sub-groups of sample sets, and determine differences between experimental groups. PCA linearly transforms and reduces the high dimensionality dataset into latent variables – specifically principal components (PCs) – to explain the variability in the dataset. Each PC is a combination of metabolite features that contributed most to the clustering of samples.

To further visualize the dataset and identify specific differences between cohorts, we used partial least-squares discriminant analysis (PLS-DA), variable importance in projection scores (VIP) and volcano plot analysis. PLS-DA is similar to PCA but is a supervised statistical analysis that is utilized to seek out differences between cohorts and reveal metabolites that are contributing to the separation between cohorts. PLS-DA is especially useful to this study because it assigns VIP scores to metabolites that are the most important in discriminating between cohorts. Volcano plot analysis was also utilized to identify significantly upregulated and downregulated metabolites with a significance level of 0.05 and false discovery rate (FDR) corrections were applied to correct for multiple comparisons. Specific metabolite features of interest identified by VIP scores and volcano plot analysis were then matched to metabolite identities and metabolic pathways using MetaboAnalyst.

## Supplementary Material

Supplementary information
