# Supplementary material for: Effects of mechanical stimulation on metabolomic profiles of SW1353 chondrocytes: shear and compression
Source: Biol Open. 2022 Feb 3;11(1):bio058895. doi: 10.1242/bio.058895 (PMC8822358; doi:10.1242/bio.058895)
Supplement: Supplementary information [file biolopen-11-058895-s1.pdf]

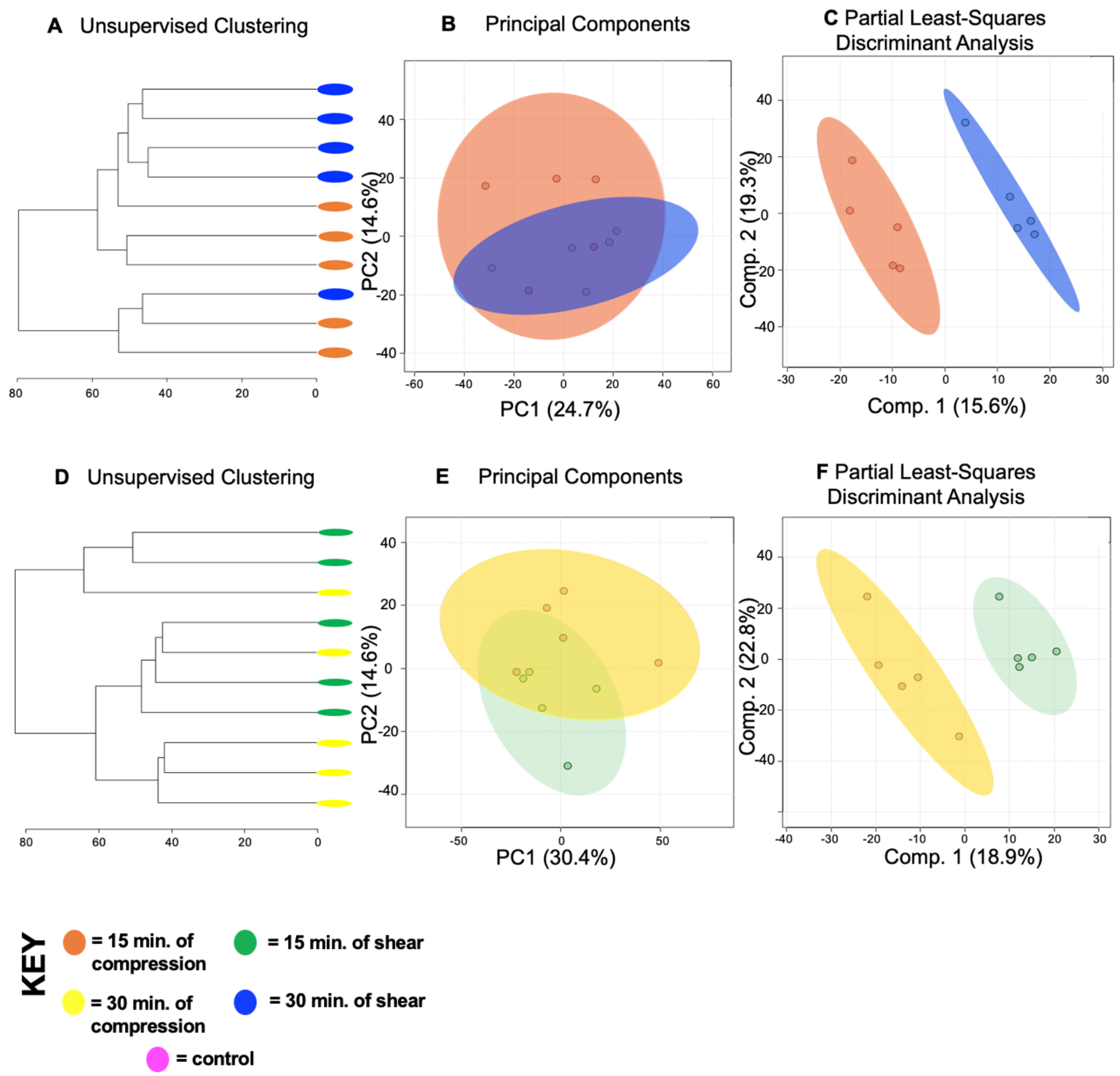

Fig. S1. Metabolic profiles of differing forces with different exposure times vary. Metabolomic profiles of chondrocytes exposed to 15 minutes of compression and 30 minutes of shear forces display distinct metabolomic profiles (A). Samples exposed to different forces for differing amounts of time somewhat separate into clusters by HCA as illustrated in the dendrogram. (B) PCA displays clear clustering of samples within their respective cohorts: chondrocytes exposed to compression for 15 minutes (orange) and chondrocytes exposed to shear forces for 30 minutes (blue). PCA is shown as a scatterplot of the first two PCs (PC1 and PC2), which account for 24.7% and 14.6% of the overall variation in the dataset respectively. (C) PLS-DA finds clear separation between samples. PLS-DA is visualized as a scatterplot of the top two components, which account for 15.6% and 19.3% of the overall variation in the

dataset. Additionally, metabolomic profiles of chondrocytes exposed to 30 minutes of compression and 15 minutes of shear force are distinct from each other. (D) HCA illustrates that groups somewhat separate. (E) PCA displays overlap of samples between chondrocytes exposed to different forces and exposure times. PC1 and PC2 account for 30.4% and 14.6% of the overall variation in the dataset respectively. (F) PLS-DA finds clear separation between samples. The two components account for 18.9% and 22.8% of variation in the dataset.

Table S1.

Click here to download Table S1

Table S2.

Click here to download Table S2

Biology Open • Supplementary information
